# Exogenous surfactant for lung contusion causing ARDS: A systematic review of clinical and experimental reports

**DOI:** 10.1111/crj.13776

**Published:** 2024-05-22

**Authors:** Tomáš Merkl, David Astapenko, Radek Štichhauer, Antonín Šafus, Tomáš Dušek, Jiří Kotek, David Řehák, Petr Lochman

**Affiliations:** ^1^ Military Faculty of Medicine, Department of Military Surgery University of Defence Hradec Kralove Czech Republic; ^2^ Department of Pediatric Surgery and Traumatology, University Hospital Hradec Kralove, Faculty of Medicine Hradec Kralove Charles University Prague Czech Republic; ^3^ Faculty of Medicine in Hradec Kralove Charles University Prague Czech Republic; ^4^ Department of Anesthesiology, Resuscitation and Intensive Medicine, University Hospital Hradec Kralove, Faculty of Medicine Hradec Kralove Charles University Prague Czech Republic; ^5^ Faculty of Health Studies Technical University in Liberec Liberec Czech Republic; ^6^ Department of Surgery, University Hospital Hradec Kralove, Faculty of Medicine Hradec Kralove Charles University Prague Czech Republic

**Keywords:** exogenous surfactant, lung contusion, pulmonary contusion, pulmonary surfactant, surface‐active agents

## Abstract

This systematic review aimed to summarize the available data on the treatment of pulmonary contusions with exogenous surfactants, determine whether this treatment benefits patients with severe pulmonary contusions, and evaluate the optimal type of surfactant, method of administration, and drug concentration. Three databases (MEDline, Scopus, and Web of Science) were searched using the following keywords: pulmonary surfactant, surface‐active agents, exogenous surfactant, pulmonary contusion, and lung contusion for articles published between 1945 and February 2023, with no language restrictions. Four reviewers independently rated the studies for inclusion, and the other four reviewers resolved conflicts. Of the 100 articles screened, six articles were included in the review. Owing to the limited number of papers on this topic, various types of studies were included (two clinical studies, two experiments, and two case reports). In all the studies, surfactant administration improved the selected ventilation parameters. The most frequently used type of surfactant was Curosurf® in the concentration of 25 mg/kg of ideal body weight. In most studies, the administration of a surfactant by bronchoscopy into the segmental bronchi was the preferable way of administration. In both clinical studies, patients who received surfactants required shorter ventilation times. The administration of exogenous surfactants improved ventilatory parameters and, thus, reduced the need for less aggressive artificial lung ventilation and ventilation days. The animal‐derived surfactant Curosurf® seems to be the most suitable substance; however, the ideal concentration remains unclear. The ideal route of administration involves a bronchoscope in the segmental bronchi.

## Graphical abstract

This systematic review aimed to summarize the available data on the treatment of pulmonary contusions with exogenous surfactants. In all the studies, surfactant administration improved the selected ventilation parameters. The most frequently used type of surfactant was Curosurf® in the concentration of 25 mg/kg. Administration of a surfactant by bronchoscopy into the segmental bronchi was the preferable way of administration. However, there are not yet enough studies to draw clear conclusions.

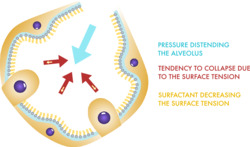

## 1 INTRODUCTION

**FIGURE 1:**
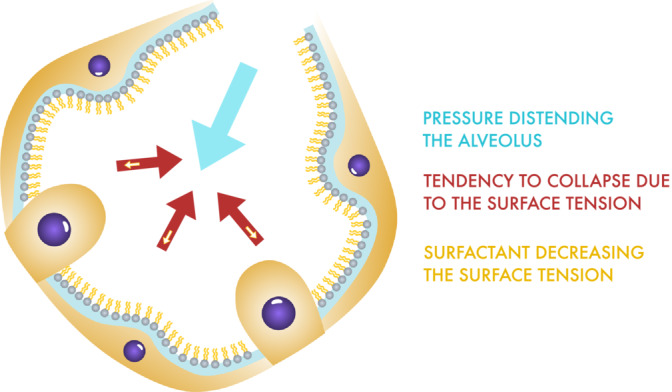
Surfactant preventing collapse of alveolus

Severe pulmonary contusion is a serious disease that complicates the healing process in patients with polytrauma due to acute respiratory distress syndrome (ARDS). It prolongs the length of stay in the intensive care unit (ICU) and increases mortality.^1^, ^2^, ^3^, ^4^ A lung contusion is an injury to the lung parenchyma without laceration of the lung tissue or vascular injury.^2^, ^5^ Most commonly, lung contusions occur in everyday life due to car accidents, falls from heights, sports injuries, and assaults.^2^, ^4^, ^6^, ^7^, ^8^, ^9^ It is also the most common injury accompanying chest trauma occurring 17–75% of cases.^2^, ^10^, ^11^, ^12^, ^13^, ^14^, ^15^, ^16^, ^17^, ^18^ Its pathophysiology results from alveolar space edema, bronchiolar obstruction by mucus plugs, and surfactant degradation. Surfactants normally decrease the surface tension of alveoli.^19^ If there is for some reason reduction in the amount of active surfactant, it leads to pulmonary atelectasis and ventilation‐perfusion mismatch.^2^, ^20^ Patients with traumatic ARDS have a higher risk of pneumonia and respiratory failure than those without pulmonary contusions.^3^, ^7^, ^11^, ^15^, ^16^ Another difference from ARDS caused by infection or aspiration is that patients with ARDS secondary to trauma are usually younger and effective interventions at an early stage of onset can lead to better outcomes and good patient prognosis.^21^ Supportive therapy is the mainstay of treatment for lung contusions with ARDS.^2^, ^7^, ^13^, ^22^ If uncomplicated, patients usually improve within 5–7 days, whereas if complicated, it may take even up to 14 days for patients to improve.^1^, ^3^, ^13^ Sometimes, they require a prone position and even extracorporeal membrane oxygenation (ECMO).^2^, ^6^, ^7^, ^23^, ^24^ Previously, the idea of treating this condition with exogenous surfactants has been suggested. There are three groups of surfactants: synthetic surfactants, surfactants produced by the lavage of bovine lungs, and surfactants obtained by the centrifugation of minced porcine or bovine lung tissue^19^, ^25^, ^26^, ^27^, ^28^ (Figure 1).

## 2 AIM

This systematic review aimed to summarize the available data on the treatment of pulmonary contusions with exogenous surfactants and whether this treatment could benefit patients with severe pulmonary contusions.

Further aims were to evaluate the optimal type of surfactant for this treatment, the method of administration, and the optimal drug concentration.

## 3 METHODOLOGY

The methodology followed the standard guidelines outlined in the Preferred Reporting Items for Systematic Reviews and Meta‐Analyses statement.^29^

### 3.1 Search strategy and article selection

The MedLine, Scopus, and Web of Science databases were searched using the following keywords: pulmonary surfactant, surface‐active agents, exogenous surfactant, pulmonary contusion, and lung contusion from 1945 to February 2023, with no language restriction.

Eligible studies were those containing treatment of lung contusions with an exogenous surfactant with full text available. All administered surfactants were included. Because of the limited number of studies on this topic, both clinical and experimental studies were included.

Studies discussing the treatment of extrapulmonary ARDS with exogenous surfactant were not included.

Four reviewers independently screened the titles and abstracts of all the relevant articles. Subsequently, the other four reviewers resolved conflicts during the initial screenings.

## 4 RESULTS

**FIGURE 2:**
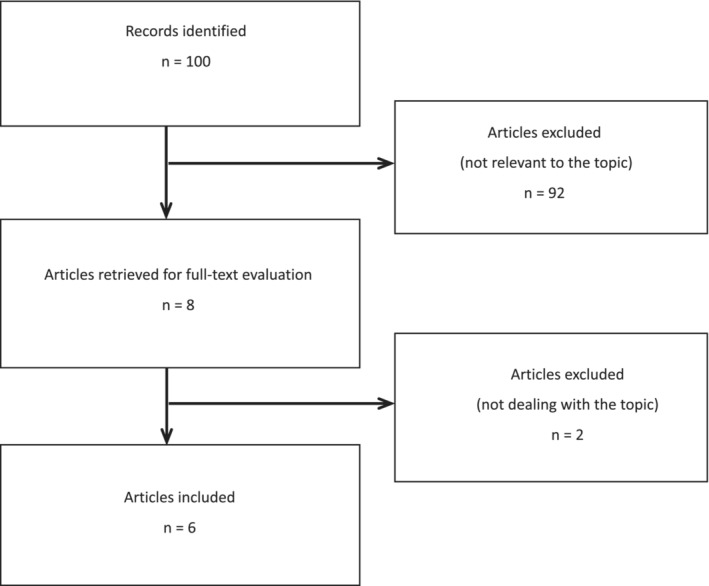
Flowchart of literature search

Of the 100 articles screened, 54 remained after the elimination of duplicates. We reviewed the full texts of eight studies. Two potentially relevant studies were excluded because of lack of full‐text availability. Six studies met our inclusion criteria. We included two clinical studies involving 60 patients, two experiments involving 70 animals (20 pigs and 50 rats), and two case reports involving two patients. A total of 94 studies were excluded. A flowchart of the literature search is shown in Figure 2.

### 4.1 Surfactant type and concentration

Porcine surfactant Curosurf® was used three times, bovine surfactant Alveofact® was used once, and in two studies, there was no further specification of the surfactant type. Concentrations from 2 to 200 mg/kg ideal body weight were used. The most commonly used concentration was 25 mg/kg in two cases (Table 1).

**TABLE 1:** Type and concentration of surfactant

	Type	Concentration
Tsangaris et al.^30^	Alveofact (bovine surfactant)	200 mg/kg
Strohmaier et al.^31^	Curosurf	25 mg/kg
Keskin et al.^5^	Not specified	100 mg/kg
Marraro et al.^18^	Curosurf	1200 mg for single dose
Sklienka et al.^32^	Curosurf	25 mg/kg
Schulz et al.^33^	Bovine surfactant	2 mg/kg

### 4.2 Surfactant administration method

In five cases, the surfactant was administered via bronchoscopy. In four cases, it extended to the segmental bronchi and, in one case, to the main bronchi. In one study, the route of administration was not specified, and the surfactant was administered via the trachea.

### 4.3 Change in ventilation parameters after surfactant administration

In all studies, the selected ventilatory parameters (FiO_2_, oxygenation index, PaO_2_/FiO_2_ ratio, and lung compliance) improved in all subjects after surfactant administration (Table 2).

**TABLE 2:** Ventilation parameters after surfactant administration

	PaO_2_/FiO_2_ ratio	Oxygenation index	FiO_2_	Lung compliance
Tsangaris et al.^30^	Gradient increase in: 6 h by 34 mmHg 12 h by 51 mmHg 24 h by 79 mmHg 48 h by 46 mmHg 72 h by 33 mmHg	Not specified	Decrease from 79% to 50%	Improvement from 30 ml/cm H2O to 36 ml/cm H_2_O
Strohmaier et al.^31^	After 4 h group with surfactant improved in the oxygenation index (parameters not specified)	Not specified	Not specified	After 8 h normalization of parameters
Keskin et al.^5^	Not specified	Not specified	Not specified	Not specified
Marraro et al.^18^	Significant improvement after 36 h	Significant improvement after 36 h	Significant improvement after 36 h	Not specified
Sklienka et al.^32^	Improvement after 1 h (parameters not specified)	Not specified	Not specified	Not specified
Schulz et al.^33^	Significant improvement after 24 h (from 50 to 750 mmHg)	Not specified	Reduction to 40% after 8 h	Not specified

### 4.4 Artificial ventilation days

In two clinical trials that examined artificial ventilation days, patients receiving surfactants required a shorter ventilation time (Table 3).

**TABLE 3:** Artificial ventilation days

	Artificial ventilation days
Tsangaris et al^30^	Surfactant: 5.6 (±2.6) Control: 8.1 (±2.4)
Strohmaier et al.^31^	Not specified
Keskin et al.^5^	Not specified
Marraro et al.^18^	Surfactant: 5.05 (±1.21) Control: 11.5 (±2.4)
Sklienka et al.^32^	54 days
Schulz et al.^33^	Died on 18th day

## 5 DISCUSSION

Severe pulmonary contusion is a serious condition that complicates the healing of patients with polytrauma. It is an injury to the lung parenchyma without laceration of the lung tissue or vascular injury and frequently results in ARDS.^2^, ^5^ Supportive care is the mainstay of treatment.^2^, ^7^, ^13^, ^22^ These include adequate oxygenation, analgesia, airway suction and toileting, positional therapy, early mobilization, and respiratory rehabilitation.^1^, ^2^, ^12^ Patients with severe pulmonary contusions require mechanical pulmonary ventilation.^2^, ^7^, ^13^, ^16^ Previously, the idea of treating this condition with exogenously administered surfactant has been suggested.^5^, ^18^, ^30^, ^31^, ^32^, ^33^

Pulmonary surfactants are mixtures of lipids and proteins that are either partially hydrophilic or partially hydrophobic. It decreases the surface tension and facilitates ventilation of the lungs. Produced by type II pneumocytes, they are present on the surface of the entire respiratory epithelium of the alveoli and respiratory bronchioles.^25^, ^26^, ^34^ A wide variety of exogenous surfactants can be used to treat ARDS, ranging from synthetically produced to animal‐derived surfactants. The future of surfactant therapy appears to involve a synthetic route; however, it is still not possible to fully optimize its production. The reason for this synthetic route is the high cost of animal surfactants.^35^ Unfortunately, synthetic surfactants do not yet reach the efficiency of animal derived surfactants.^36^ Previously, all the authors chose animal‐derived surfactants. Two products were used: Curosurf®, which is produced by homogenization and centrifugation of the porcine lung, and Alveofact®, which is obtained by lavage of bovine lungs. The majority of the authors chose Curosurf® probably due to its relatively easy availability and the longer experience with this product, as it is commonly used in neonates with respiratory distress syndrome and is therefore already registered as an approved therapeutic agent for use in patients.^37^, ^38^ The optimal concentration of the surfactant to be administered remains to be determined. The opinions of the authors varied widely, ranging from 2 mg/kg ideal body weight to 200 mg/kg. The standard dosage of surfactant in children with RDS is between 100 and 200 mg/kg, which would require a significant dose of this relatively expensive drug in an adult. It is desirable to deliver sufficient concentration to improve the patient's ventilatory parameters. However, given the high cost of surfactants, it is necessary to determine the lowest effective concentration to reduce the cost of patient treatment. This is made possible by the assumption that, unlike the newborn with RDS, the endogenous surfactant persists at least partially in the adult lungs with ARDS. The most common concentration was 25 mg/kg, which appeared to be the most cost‐effective dose.

There are various approaches for surfactant administration, including bronchoscopy, tracheal tube, nasogastric tube, and nebulization.^25^, ^26^ The administration of a surfactant as an aerosol is an interesting idea; however, it faces technical issues. The advantage of this route is that intubation of the patient is not necessary; however, for our purposes, this advantage becomes meaningless because artificial ventilation is presumptive in severe pulmonary contusion. The administration of surfactant via an endotracheal tube does not permit control of the distribution of the surfactant, which may not reach the regions of the lungs that are most severely affected.^39^ Thus, from our point of view, administration by bronchoscope is preferable. Bronchoscopic administration ensured even distribution of surfactants across the lungs. Ruaro et al.^39^ claim that by using bronchoscopic lavage, they reduced the volume of surfactant required for the treatment by a factor of five times compared to using a bolus of surfactnat administered bronchoscopically. Differences among the authors of reviewed papers were observed in place of administration. Keskin et al.^5^ administered a surfactant intratracheally, Marraro et al.^18^ administered a surfactant into the main bronchi, and the remaining authors reported administration into the segmental bronchi.^30^, ^31^, ^32^, ^33^ Although all the authors reported positive results, we believe that administration to the segmental bronchi will ensure a more even distribution of surfactants and, therefore, a better therapeutic effect. However, this conclusion is based only on the results of a few studies. So far, no comparative study has been done to determine the ideal route of surfactant administration.

The main reason for surfactant administration in lung contusions is to improve ventilatory parameters. This has to the need for less aggressive ventilation and earlier extubation.^18^, ^30^ Excessive invasive ventilation can lead to barotrauma and paradoxical deterioration of respiratory function.^16^ Thus, protective ventilation is necessary, where the tidal volume is calculated as 4–8 ml/kg of the ideal body weight.^6^, ^7^, ^16^, ^23^ However, this strategy may be inadequate to meet the needs of patients, with polytrauma and a high oxygen demand. Therefore, surfactant therapy is considered to be beneficial. The authors of the reviewed articles examined several parameters, such as oxygenation index, PaO_2_/FiO_2_ ratio, FiO_2_, and lung compliance. In all the studies, the group in which the surfactant was administered showed an improvement in the monitored parameters and thus ensured sufficient oxygenation, even with a less aggressive ventilatory setup. Thus, patients with severe lung contusions benefit from exogenous surfactant administration.

Another important parameter for evaluating the benefits of surfactant treatment is the number of days on the ventilator. This parameter has been investigated in two clinical trials.^18^, ^30^ Tsangaris et al.^30^ reported a reduction in the required ventilation time by 3 days. Marraro et al.^18^ reported a reduction in this time even by 6.5 days compared to the control group. This is of major importance to patients in terms of the complications associated with artificial lung ventilation, sedation, and positioning. Invasive ventilation increases the risk of barotrauma and is the greatest risk factor of lung parenchymal infections.^1^, ^3^, ^8^, ^40^ Pneumonia in pulmonary contusions increases the mortality rate.^8^ Mechanical ventilation also increases mortality and morbidity in patients with ARDS.^23^

Although this is an interesting topic, not many studies dealt with this treatment so far. The available data are difficult to compare with each other owing to the diversity of the types of studies and subjects studied; however, the available data suggest that this treatment seems promising for the future. Further studies on this topic are required to complete the current knowledge and add important information, such as the ideal concentration of surfactants.

## 6 CONCLUSION

Concerning the reviewed literature, the administration of exogenous surfactants improves ventilatory parameters, thus reducing the need for less aggressive artificial lung ventilation and reduced ventilation days. Thus, surfactants play a role in preventing the complications associated with artificial pulmonary ventilation. The animal‐derived surfactant Curosurf® seems to be the most suitable substance; however, the ideal concentration remains unclear. The ideal route of administration involves a bronchoscope in the segmental bronchi. Unfortunately, not enough papers have been published on this topic to draw clear conclusions. Therefore, more randomized clinical trials devoted to patients with ARDS due to lung contusion are required in the future.

## CONFLICT OF INTEREST

The authors declare no conflict of interest.

## AUTHOR CONTRIBUTIONS

Tomáš Merkl designed the review methods, performed the literature search, collected the data, analyzed the data, and wrote the paper; David Astapenko analyzed the data and wrote and edited the manuscript; Radek Štichhauer analyzed the data; Antonín Šafus collected the data; Tomáš Dušek collected the data; Jiří Kotek collected the data; David Řehák designed and created the illustration; and Petr Lochman analyzed the data.

## ETHICS STATEMENT

Since this is a systematic review, ethical approval is not required.

## Data Availability

The data that support the findings of this study are available in the following resources available in the public domain: PubMed (https://pubmed.ncbi.nlm.nih.gov/), Web of Science (https://www.webofscience.com/), and Scopus (https://www.scopus.com/).
